# B-1a cells protect mice from sepsis-induced acute lung injury

**DOI:** 10.1186/s10020-018-0029-2

**Published:** 2018-05-29

**Authors:** Monowar Aziz, Yasumasa Ode, Mian Zhou, Mahendar Ochani, Nichol E. Holodick, Thomas L. Rothstein, Ping Wang

**Affiliations:** 1 Center for Immunology and Inflammation, The Feinstein Institute for Medical Research, 350 Community Dr, Manhasset, NY 11030 USA; 2 Center for Oncology and Cell Biology, The Feinstein Institute for Medical Research, Manhasset, New York, 11030 USA; 3 Department of Surgery and Molecular Medicine, Donald and Barbara Zucker School of Medicine at Hofstra/Northwell, Manhasset, New York, 11030 USA; 4 Present Address: Western Michigan University Homer Stryker M.D. School of Medicine, 1000 Oakland Drive, Kalamazoo, MI 49008 USA

**Keywords:** B-1a cells, Sepsis, Acute lung injury, Inflammation, Neutrophils, IL-10

## Abstract

**Background:**

Sepsis morbidity and mortality are aggravated by acute lung injury (ALI) or acute respiratory distress syndrome (ARDS). Mouse B-1a cells are a phenotypically and functionally unique sub-population of B cells, providing immediate protection against infection by releasing natural antibodies and immunomodulatory molecules. We hypothesize that B-1a cells ameliorate sepsis-induced ALI.

**Methods:**

Sepsis was induced in C57BL/6 mice by cecal ligation and puncture (CLP). PBS or B-1a cells were adoptively transferred into the septic mice intraperitoneally. After 20 h of CLP, lungs were harvested and assessed by PCR and ELISA for pro-inflammatory cytokines (IL-6, IL-1β) and chemokine (MIP-2) expression, by histology for injury, by TUNEL and cleaved caspase-3 for apoptosis, and by myeloperoxidase (MPO) assay for neutrophil infiltration.

**Results:**

We found that septic mice adoptively transferred with B-1a cells significantly decreased the mRNA and protein levels of IL-6, IL-1β and MIP-2 in the lungs compared to PBS-treated mice. Mice treated with B-1a cells showed dramatic improvement in lung injury compared to PBS-treated mice after sepsis. We found apoptosis in the lungs was significantly inhibited in B-1a cell injected mice compared to PBS-treated mice after sepsis. B-1a cell treatment significantly down-regulated MPO levels in the lungs compared to PBS-treated mice in sepsis. The protective outcomes of B-1a cells in ALI was further confirmed by using B-1a cell deficient CD19^−/−^ mice, which showed significant increase in the lung injury scores following sepsis as compared to WT mice.

**Conclusions:**

Our results demonstrate a novel therapeutic potential of B-1a cells to treat sepsis-induced ALI.

## Background

Based on the Third International Consensus Definitions for Sepsis and Septic Shock (Sepsis-3), sepsis is defined as “life-threatening organ dysfunction caused by a dysregulated host response to infection” (Singer et al. 2016). In the United States, there are approximately 1 million cases of sepsis annually, with a mortality rate up to 40% (Vincent et al. 2014; Martin et al. 2006; Aziz et al. 2013). The lungs are particularly susceptible to injury during sepsis, and more than 50% of patients with sepsis develop acute lung injury (ALI) or acute respiratory distress syndrome (ARDS) (Sevransky et al. 2009; Gu et al. 2014). The pathophysiology of sepsis-induced ALI is less well understood. Antibiotics and supportive measures are the only treatments available for patients with sepsis and ALI, and these measures have limited impact on the high mortality rates of sepsis.

Immune cells recognize pathogen-associated molecular patterns (PAMPs) via their toll-like receptors (TLRs) to exaggerate “cytokine storm”, which trigger inflammation and impair tissue function during sepsis (Aziz et al. 2013; Barton and Medzhitov 2003; Foster and Medzhitov 2009). Neutrophil infiltration in lungs is a major pathophysiological hallmark of ALI. Uncontrolled migration of neutrophils into lungs leads to exaggerated production of cytokines, chemokines, myeloperoxidase (MPO), reactive oxygen species (ROS), nitric oxide (NO), and neutrophil extracellular traps (NETs) causing unrestrained inflammation, lung dysfunction and death (Aziz et al. 2013; Grommes and Soehnlein 2011; Abraham 2003; Lee and Downey 2001; Brinkmann et al. 2004; Kaplan and Radic 2012; Delgado-Rizo et al. 2017). Thus, regulating the exaggerated function of neutrophils and their uncontrolled infiltration into lungs serves as an effective therapeutic tool in ALI. The early onset of pro-inflammatory cytokine storm, often contributing to the lung injury in sepsis, can be reversed by the actions of anti-inflammatory cytokines such as interleukin (IL)-10 (Cinel and Opal 2009; Kono et al. 2006). We recently demonstrated the beneficial role of IL-10 producing B-1a cells in sepsis by controlling the systemic levels of pro-inflammatory cytokines, chemokines and bacterial loads (Aziz et al. 2017), while their role in ALI remained unknown. Elucidation of the novel role of B-1a cells in lungs during sepsis will not only improve our understanding of ALI pathophysiology, but also help us to develop effective therapeutics against ALI.

In mouse, B cells consist of various subpopulations, which include follicular (FO), marginal zone (MZ) and B-1 B cells (Aziz et al. 2015). The role of FO and MZ B cells collectively known as B-2 cells in the early immune response and inflammatory cytokine production during sepsis has been demonstrated previously (Kelly-Scumpia et al. 2011; Honda et al. 2016). B-1 cells comprising a minor portion of the total B cells in mice display unique features in terms of their phenotype, localization, development, signaling and function (Aziz et al. 2015; Martin and Kearney 2001; Kantor et al. 1992). The surface phenotype of murine B-1 cells is B220^lo^, IgM^hi^, IgD^lo^, CD23^−^, CD19^hi^ and CD43^+^ (Aziz et al. 2017; Aziz et al. 2015; Kantor et al. 1992). B-1 cells can be further divided into B-1a and B-1b cells, depending on their surface expression of CD5 (Kantor et al. 1992; Berland and Wortis 2002). B-1a cells are predominantly localized in the peritoneal cavity; however, a small portion of B-1a cells can also be found in the respiratory tract, intestinal tissues, lymph nodes, spleen and bone marrow (Aziz et al. 2015; Yenson and Baumgarth 2014). B1a cells can secrete large amounts of natural IgM and IgA that are capable of recognition and clearance of invading pathogens (Aziz et al. 2015; Grönwall et al. 2012; Vas et al. 2012). Natural antibodies have antigen specificity for a number of microbial epitopes such as phospholipids and lipopolysaccharides (LPS) (Grönwall et al. 2012; Vas et al. 2012). Murine B-1a cells are known to produce ample amount of IL-10 and granulocyte macrophage colony stimulating factor (GM-CSF), which attenuate excessive inflammation during sepsis (Aziz et al. 2017; Aziz et al. 2015; Rauch et al. 2012).

Recent findings demonstrate an active role of B-1a cells for protection against lung infection caused by influenza virus (Baumgarth et al. 1999; Baumgarth et al. 2000; Choi and Baumgarth 2008). During influenza virus infection B-1a cells migrate from serosal cavities to the lungs, where they secrete natural Abs and other immunomodulatory molecules to protect rodents against influenza virus infection (Baumgarth et al. 2000; Choi and Baumgarth 2008). Consistently, in various animal models of ALI initiated by direct instillation of LPS, *E. coli* or *S. pneumoniae,* B-1a cells were shown to migrate from the pleural cavity to the lung parenchymal tissues, where they secrete GM-CSF and IgM to protect rodents against ALI (Weber et al. 2014). A recent study has demonstrated that due to the loss of function of natural IgM as secreted from the B-1a cells could be the cause of poor prognostic outcomes of lung infection in aged animals (Holodick et al. 2016). The beneficial role of B-1a cells in lungs was shown in virus and bacterial infections, as well as in young over old mice with *S. pneumoniae* infection, indicating that these cells play a pivotal role in lung diseases. Nonetheless, their role in sepsis-induced ALI remains unknown.

In the current study, we aimed to study the role of B-1a cells in ALI during sepsis. Our study for the first time revealed the protective role of B-1a cells against sepsis-induced ALI by controlling exaggerated inflammation and infiltration of neutrophils in lungs. Thus, B-1a cells could represent a promising therapeutic in sepsis-induced ALI.

## Methods

### Animals

Wild-type (WT) C57BL/6 mice obtained from Taconic (Albany, NY) and B6.129P2(C)CD19^*tm1(cre)cgn*^/J (CD19^−/−^) mice obtained from The Jackson Laboratory were housed in a temperature and light controlled room and fed a standard laboratory diet. For all experiments male 8- to 10-week-old 21–28 g of body weight (BW) mice were used. Animals were randomly assigned to sham, vehicle control and B-1a cell treatment groups. Number of animals estimated in each group was based on our previous study on an animal model of sepsis (Aziz et al. 2017). All animal protocols were approved by our Institutional Animal Care and Use Committee.

### Murine model of polymicrobial sepsis

Mice were anesthetized with 2% isofluorane inhalation and underwent cecal ligation and puncture (CLP). A 2-cm incision was made to the abdominal wall, and the cecum was exposed and ligated 0.5 cm from the tip with 4–0 silk suture. A 22-gauge needle was used to make one puncture through and through to the distal cecum, extruding a small amount of fecal contents. The cecum was replaced into the abdominal cavity, and the exposed abdominal wall was closed in two layers with running 4–0 silk suture. In sham-operated mice only laparotomy was performed, but their cecum was not ligated and punctured. Animals were resuscitated with 1 ml of normal saline subcutaneously. In another experiment, WT and CD19^−/−^ mice were subjected to either sham or CLP operation following the above CLP protocol.

### Adoptive transfer of murine B-1a cells

Murine B-1a cells in peritoneal washouts were stained with FITC-B220 (clone RA3-6B2), Pacific Blue-CD23 (clone B3B4), and PE-Cy5-CD5 (clone 53-7.3) obtained from BD Biosciences (San Diego, CA). B-1a cells with phenotype, CD23^−^B220^lo^CD5^int^ were sort-purified using a BD Biosciences Influx instrument. Post-sort analysis showed PerC B-1a cells to be ≥98% pure. Sort-purified B-1a cells were washed with PBS and then suspended in PBS for adoptive transfer into septic mice through intraperitoneal (*i.p.*) injection. At the time of CLP operation, 5 × 10^5^ B-1a cells suspended in 150 μl of PBS were delivered into the peritoneal cavity and the abdominal wound was closed with running 4–0 silk suture. As vehicle negative control, 150 μl of PBS was injected into the abdomen of CLP-operated mice. The animals were allowed food and water ad libitum, and at 20 h after CLP operation and B-1a cell transfer the animals were euthanized and lungs were collected for various ex vivo analyses.

### Quantitative real-time PCR assay

Total RNA was extracted from lung tissues using TRIzol reagent (Invitrogen; Carlsbad, CA) and reverse-transcribed into cDNA using reverse transcriptase enzyme (Applied Biosystems; Foster City, CA). The PCR reaction was performed in 20 μl of final volume containing 0.08 μM of forward and reverse primer, 2 μl of 10–20× diluted original cDNA, and 10 μl SYBR Green PCR Master Mix (Applied Biosystems) using Applied Biosystems 7300 real-time PCR machine. Mouse β-actin served as an internal control gene for normalization. Relative expression of mRNA was represented as fold change in comparison to the sham group. The sense and anti-sense primer sequences of mouse genes are, IL-6 (NM_031168): 5’-CCGGAGAGGAGACTTCACAG-3′ and 5′-GGAAATTGGGGTAGGAAGGA-3′; IL-1β (NM_008361): 5’-CAGGATGAGGACATGAGCACC-3′ and 5’-CTCTGCAGACTCAAACTCCAC-3′; tumor necrosis factor-α (TNF-α) (NM_013693.2): *5′-AGACCCTCACACTCAGATCATCTTC-3′* and *5′-TTGCTACGACGTGGGCTACA-3′*; interferon γ (IFNγ) (NM_008337): 5’-GGCTTTGCAGCTCTTCCTC-3′ and 5’-CCAGTTCCTCCAGATATCCAA-3′; IL-10 (NM_010548): 5’-CAGCCGGGAAGACAATAA CT-3′ and 5’-GCATTAAGGAGTCGGTTAGCA-3′; MIP-2 (NM_009140): 5’-CCCTGGTTC AGAAAATCATCCA-3′ and 5’-GCTCCTCCTTTCCAGGTCAGT-3′; β-actin (NM_007393): 5’-CGTGAAAAGATGACCCAGATCA-3′ and 5’-TGGTACGACCAGAGGCATACAG-3′.

### ELISA

The lung tissue was crushed in liquid nitrogen, and approximately 50 mg of powdered tissues were dissolved in 500 μl of lysis buffer (10 mM Hepes, pH 7.4, 5 mM MgCl_2_, 1 mM DTT, 1% Triton X-100, and 2 mM each of EDTA and EGTA), and subjected to sonication on ice. Protein concentration was determined by the BioRad protein assay reagent (Hercules, CA). Equal amounts (50 μg) of proteins were loaded into respective enzyme-linked immunosorbent assay (ELISA) wells for assessment of IL-6, IL-1β, TNF-α, IFNγ, IL-10 and MIP-2 by using the kits obtained from BD Biosciences, and IgM by using the kit from Bethyl Laboratories, Inc., Montgomery, TX.

### Lung tissue histology

Formalin fixed and paraffin embedded lung tissue blocks were sectioned at 5 μm thickness and placed on glass slides. Lung tissue sections were stained with hematoxylin & eosin (H&E) and observed under a light microscope. Morphological changes were scored as nil (0), mild (1), moderate (2), or severe (3) injury based on the presence of exudates, hyperemia or congestion, infiltration of neutrophils, alveolar hemorrhage, presence of debris, and cellular hyperplasia, in a blinded fashion (Aziz et al. 2012; Hirano et al. 2015). The sums of scores of different animals were averaged and plotted on a bar graph.

### Myeloperoxidase assay

A total of 50–100 mg of liquid nitrogen-based powered lung tissues were homogenized in KPO_4_ buffer containing 0.5% hexa-decyl-trimethyl-ammonium bromide (Sigma-Aldrich, St. Louis, MO) using a sonicator with the samples placed in ice. After centrifuging, the supernatant was diluted in reaction solution which contains O-Dianisidine dihydrochloride (Sigma-Aldrich) and H_2_O_2_ (ThermoFisher Scientific, Waltham, MA) as substrate. Rate of change in optical density (∆OD) between 1 and 4 min was measured at 460 nm to calculate myeloperoxidase (MPO) activity (Aziz et al. 2012).

### TUNEL assay

The presence of apoptotic cells in lung tissue sections was determined using a terminal deoxynucleotide transferase dUTP nick end labeling (TUNEL) assay kit (Roche Diagnostics, Indianapolis, IN). Briefly, lung tissues were fixed in 10% phosphate buffered formalin and were then embedded into paraffin and sectioned at 5 μm following standard histology procedures. Lung sections were dewaxed, rehydrated and equilibrated in Tris buffered saline (TBS). The sections were then digested with 20 μg/mL proteinase K^+^ for 20 min at room temperature. The lung tissue sections were then washed and incubated with a cocktail containing terminal deoxynucleotidyl transferase enzyme and fluorescence labeled nucleotides and examined under a fluorescence microscope (Nikon Eclipse Ti-S, Melville, NY).

### Caspase-3 enzyme activity assay

The caspase-3 enzyme activity in lung tissues was assessed by a fluorimetric assay system kit (Sigma, Saint Louis, MO). Lung tissues were homogenized in liquid nitrogen, and approximately 50 mg of powdered tissues were dissolved in 500 μl of lysis buffer, which contains a cocktail of 10 mM Hepes, pH 7.4, 5 mM MgCl_2_, 1 mM DTT, 1% Triton X-100, and 2 mM each of EDTA and EGTA, and then subjected to sonication by placing the samples in ice. Protein concentration was measured by the Bio Rad protein assay reagent (Hercules). Equal amounts of proteins in a 5 μl volume were added to the 100 μl assay buffer (20 mM Hepes, pH 7.4, 5 mM DTT, 2 mM EDTA, and 0.1% CHAPS) containing 10 μM DEVD-AMC substrate molecule and the rate of changes of fluorescence intensity at 37 °C were measured at 370 nm (excitation wavelength) and 450 nm (emission wavelength) in a fluorometer (Synergy H1, BioTek, Winooski, VT). The caspase-3 enzyme activity was expressed as mM AMC/min/g of protein (Aziz et al. 2012).

### Statistical analysis

Figure preparation and data analyses were performed by using SigmaPlot 12.5 software (Systat Software Inc., San Jose, CA). Data represented in the figures are expressed as mean ± standard error (SE). One way analysis of variance (ANOVA) was used for comparison among multiple groups and the significance was determined by the Student-Newman-Keuls (SNK) test. Paired two-tailed Student’s *t*-test was applied for two-group comparisons. Significance was determined as *p* ≤ 0.05 between experimental groups.

## Results

### B-1a cells attenuate the expression of pro-inflammatory cytokines in the lungs during sepsis

**Fig. 1:**
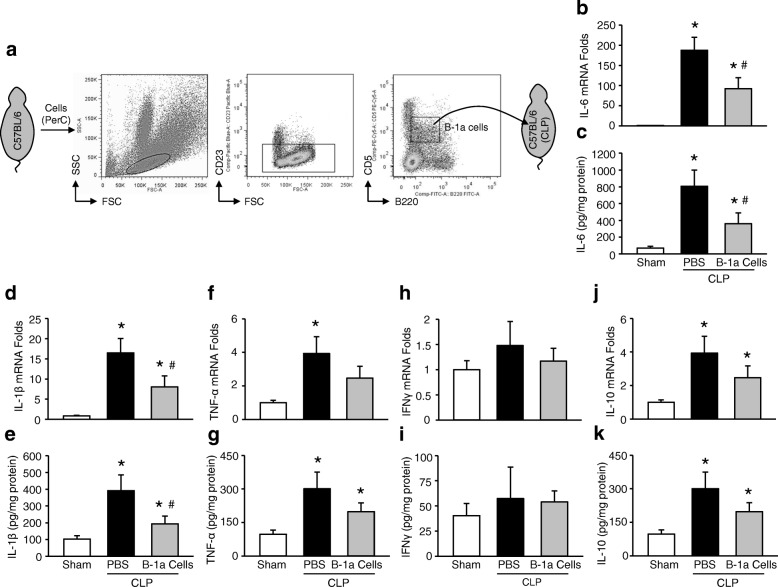
Adoptive transfer of B-1a cells attenuates lung inflammation. **a** Peritoneal washout cells isolated from healthy mice were stained with anti-mouse Pacific Blue-CD23, FITC-B220 and PE-Cy5 Abs and subjected to sort purification by using a flow cytometry-based cell sorting system. A total of 5 × 10^5^ B-1a cells suspended in 150 μl of PBS were delivered into the peritoneal cavity of CLP mice. After 20 h, lung tissue was harvested and mRNA and protein expression of **b**, **c** IL-6, **d**, **e** IL-1β, **f**, **g** TNF-α, **h**, **i** IFNγ and **j**, **k** IL-10 were assessed, respectively. Data are expressed as means ± SE (*n* = 9 mice/group) and compared by one-way ANOVA and SNK method (^*^p < 0.05 vs. sham mice; ^#^*p* < 0.05 vs. PBS-treated CLP mice). CLP, cecal ligation and puncture; IL, interleukin

Peritoneal B-1a cells were sort-purified based on CD23^−^B220^lo^CD5^int^ surface phenotype from healthy mice and then injected into mice immediately after CLP operation (Fig. 1a). At 20 h after CLP operation, lungs were harvested to assess the expression of pro- and anti-inflammatory cytokines. Expression of IL-6 and IL-1β in lung tissue from CLP mice was significantly up-regulated compared to sham-operated mice, while the adoptive transfer of B-1a cells significantly down-regulated expression of IL-6 and IL-1β by 51 and 54%, respectively at the mRNA and 55 and 51%, respectively at the protein level (Fig. 1b-e). We found significant up-regulation of the expression of TNF-α at mRNA and protein levels in the lung tissues of CLP mice, while there was a trend towards down-regulation of TNF-α expression in lungs of B-1a cell-treated CLP mice as compared to vehicle-treated CLP mice (Fig. 1f, g). We could not find significant increase of the expression of IFNγ at both mRNA and protein levels in lungs at 20 h of CLP, which could be due to the fact that its up-regulation might occur at earlier time point after CLP operation, and therefore 20 h after CLP was too late to determine its up-regulation in lung tissues (Fig. 1h, i). Similar to the patterns of expression of pro-inflammatory cytokines, we found significant up-regulation of IL-10 expression at mRNA and protein levels in the lung tissues following CLP operation as compared to sham-operated mice (Fig. 1j, k). We noticed a trend towards decreasing the expression of IL-10 in lungs of B-1a cell-treated CLP mice as compared to vehicle-treated CLP mice, reflecting the remission of inflammation after B-1a cell treatment in septic mice (Fig. 1j, k).

### Treatment of septic mice with B-1a cells attenuates lung injury scores

**Fig. 2:**
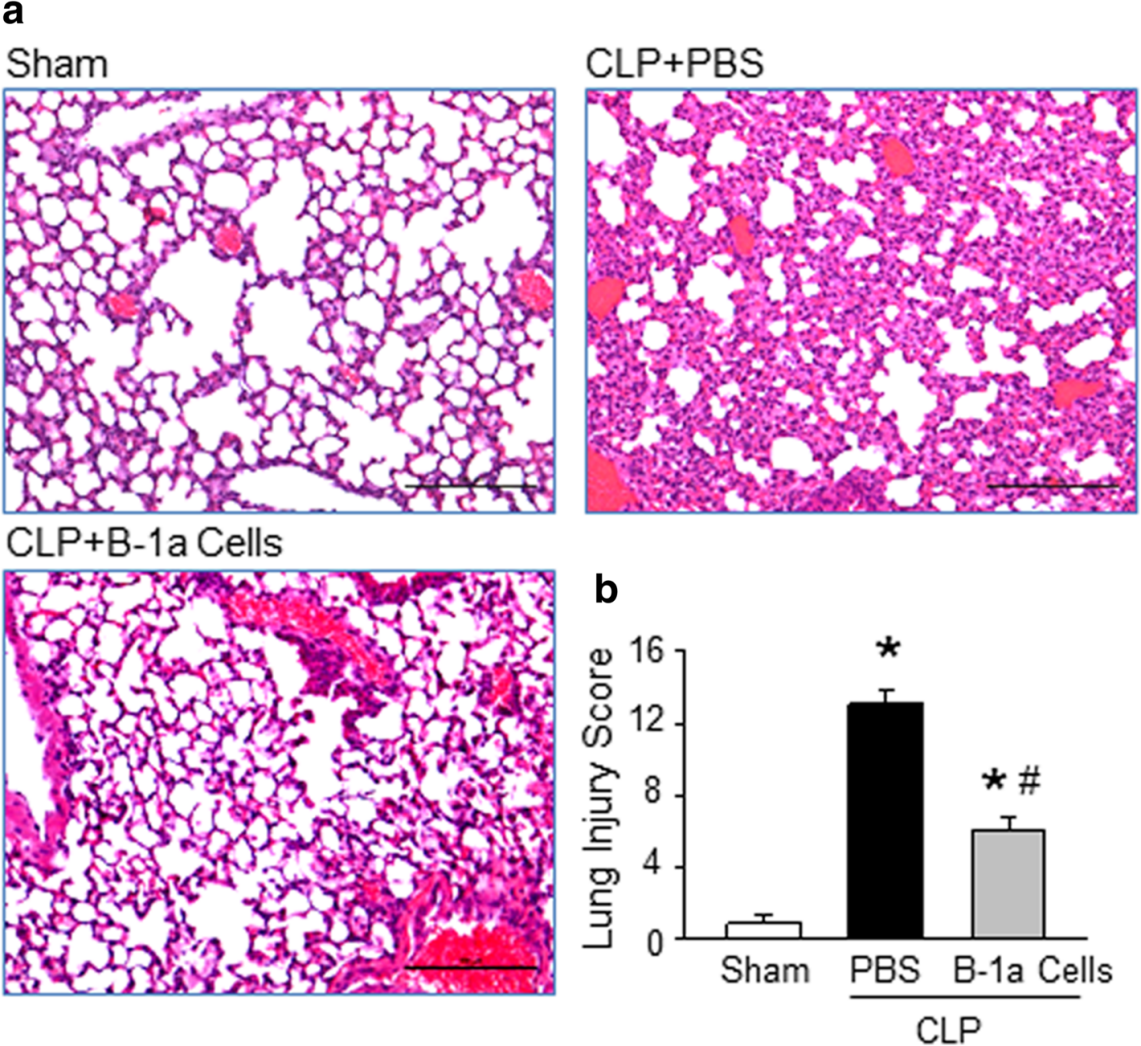
Treatment with B-1a cells improves the histopathological *score* of lung tissue damage in sepsis. **a** Lung tissue was collected after 20 h from sham-operated, and either PBS- or B-1a cell-treated CLP mice and stained with H&E. Each slide was observed under light microscopy at × 100 original magnification in a blinded fashion. Representative images for each group are shown. Scale bar, 100 μm. **b** Histological injury scores of the lungs in different groups were quantified as described in Materials and Methods. Data from three independent experiments are expressed as means ± SE (*n* = 6 mice/group) and compared by one-way ANOVA and SNK method (^*^p < 0.05 vs. shams; ^#^*p* < 0.05 vs. PBS-treated CLP mice). CLP, cecal ligation and puncture; H&E, hematoxylin and eosin

Histological images of lung tissue showed decreased levels of alveolar congestion, exudate, interstitial and alveolar cellular infiltrates, intra-alveolar capillary hemorrhages, and damage of epithelial architecture, in B-1a cell-treated CLP mice as compared to PBS-treated CLP mice (Fig. 2a). These histological changes were reflected in a significant decrease in lung tissue injury score in B-1a cell-treated mice compared to PBS-treated CLP mice by a mean value of 54% (Fig. 2b). On the other hand, the sham-operated mouse lungs showed normal histological architecture.

### B-1a cells attenuate chemokine and MPO levels in the lungs of septic mice

**Fig. 3:**
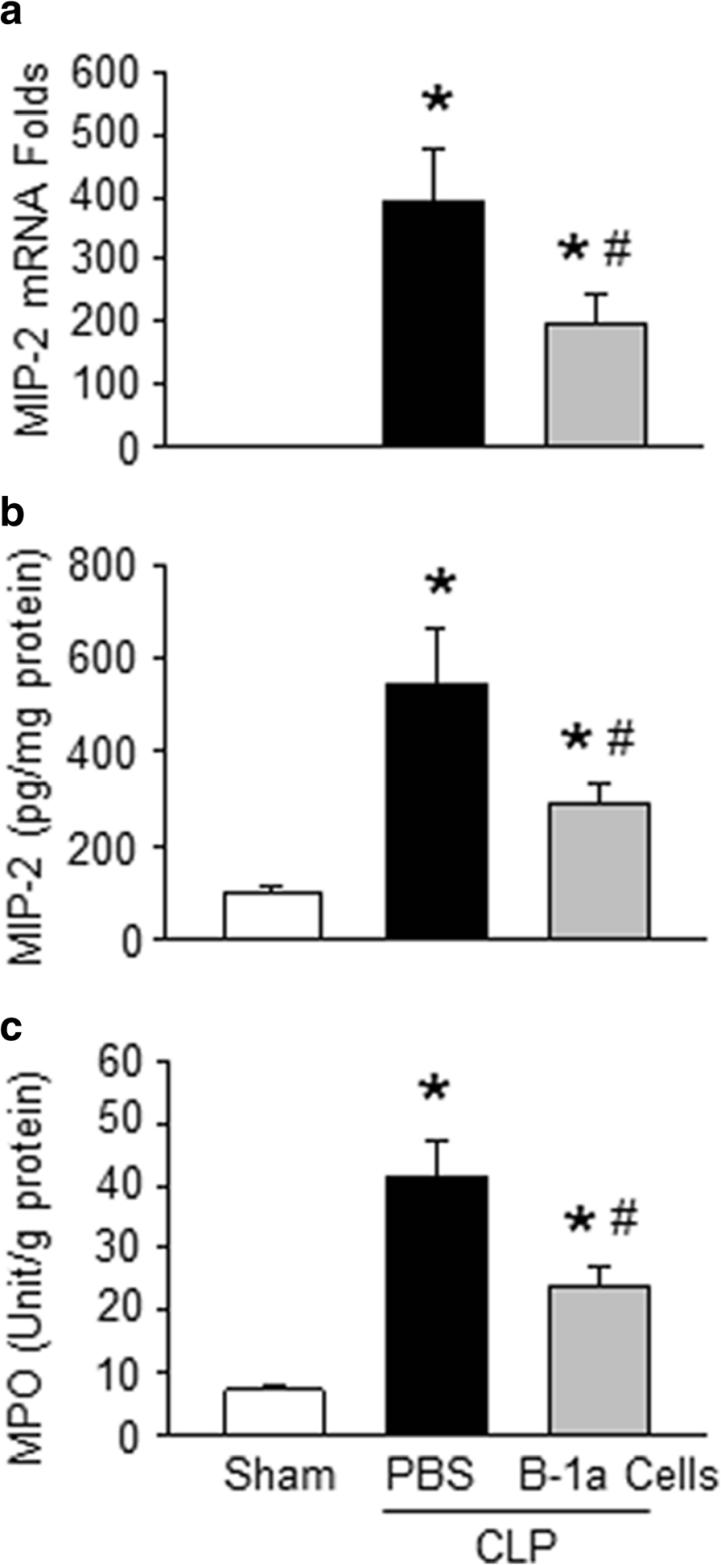
B-1a cells attenuate MIP-2 and MPO levels in lungs after sepsis. **a**, **b** At the time of CLP, mice were treated with either PBS as vehicle or 5 × 10^5^ PerC B-1a cells in 150 μl of PBS by *i.p.* injection. After 20 h, lung tissue was harvested and mRNA and protein expression of MIP-2 were assessed, respectively. **c** MPO activity in lungs of sham-operated, and PBS or B-1a cell-treated CLP mice was determined. Data are expressed as means ± SE (*n* = 9 mice/group from 3 independent experiments) and compared by one-way ANOVA and SNK method (^*^p < 0.05 vs. shams; ^#^*p* < 0.05 vs. PBS-treated CLP mice). CLP, cecal ligation and puncture; MIP-2, macrophage-inflammatory protein-2; MPO, myeloperoxidase

Chemokines such as MIP-2 play a pivotal role in the infiltration of neutrophils in lungs during sepsis (Aziz et al. 2013; Abraham 2003). In lung tissue following sepsis, we noticed significant up-regulation of MIP-2 expression compared to sham mice, while the mice treated with B-1a cells significantly reduced the expression of MIP-2 by 49 and 46%, respectively at the mRNA and protein levels compared to PBS-treated CLP mice (Fig. 3a, b). The neutrophil infiltration in lungs as measured by the amount of MPO showed significant inhibition in B-1a cell-treated mice by 41% as compared to PBS-treated mice during CLP (Fig. 3c).

### Treatment with B-1a cells attenuates apoptosis in the lung during sepsis

**Fig. 4:**
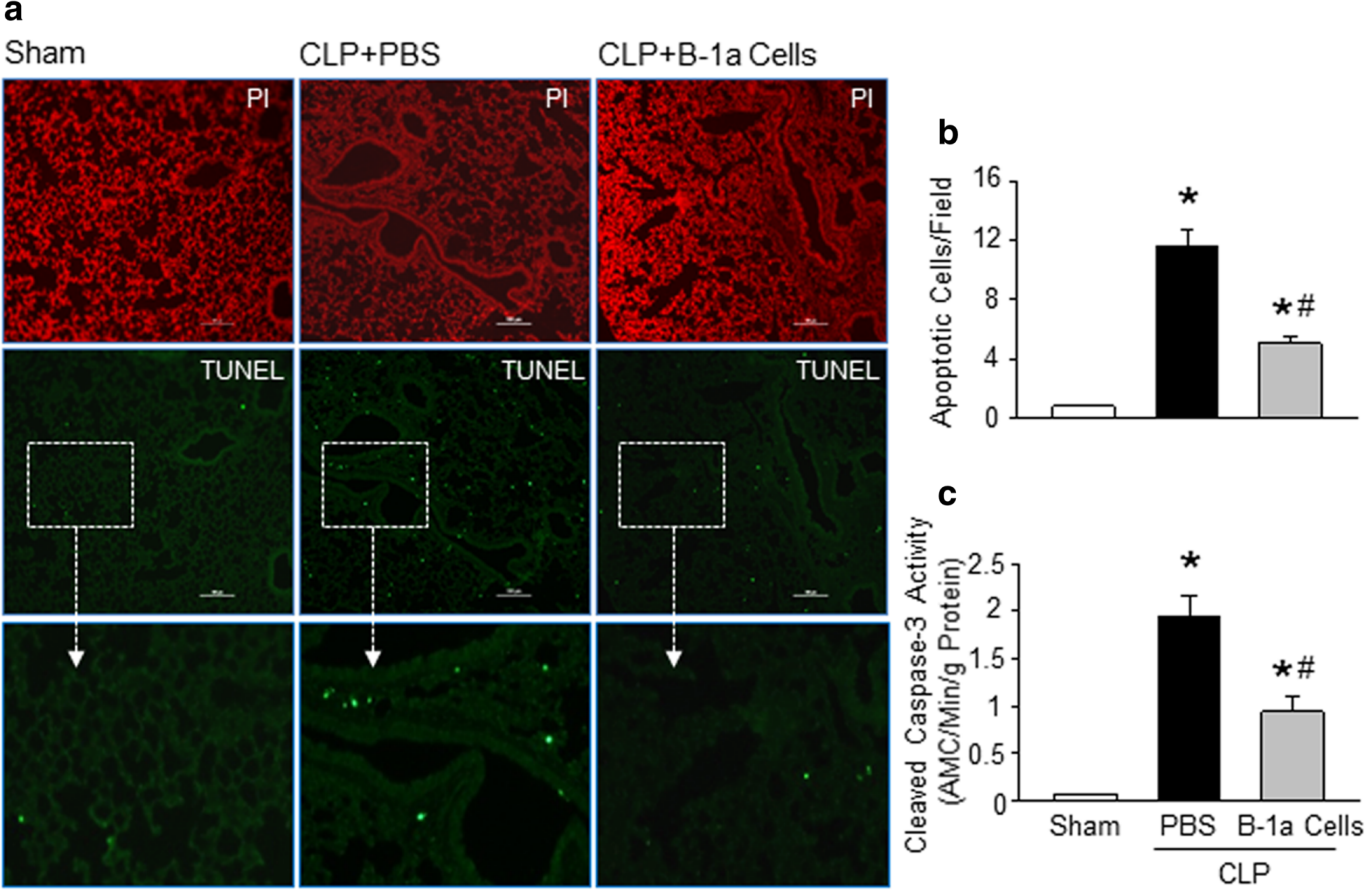
Treatment with B-1a cells attenuates apoptosis in lungs after sepsis. After 20 h of CLP, lung tissues were collected from PBS or B-1a cell treated mice. **a** Lung tissue sections were prepared for TUNEL staining shown in green, and for nuclear staining using PI shown in red. Representative images at × 100 original magnification are shown. Scale bar, 100 μm. **b** TUNEL positive apoptotic cells were counted at 18 random fields in a blinded fashion, and the average numbers of cells per field are shown. **c** Cleaved Caspase-3 activity in total lung tissues of sham-operated, and PBS or B-1a cell-treated CLP mice was determined. Data are expressed as means ± SE (*n* = 6 mice/group) and compared by one-way ANOVA and SNK method (^*^p < 0.05 vs. shams; ^#^*p* < 0.05 vs. PBS-treated CLP mice). CLP, cecal ligation and puncture; TUNEL, terminal deoxynucleotidyl transferase dUTP nick end labeling; PI, propidium iodide

Sepsis resulted in a significant increase in the number of apoptotic cells in lungs (Aziz et al. 2013; Aziz et al. 2012). Here, we noticed that the septic mice treated with B-1a cells experienced a significant decrease in the numbers of apoptotic cells by 56% compared to PBS-treated septic mice (Fig. 4a, b). Furthermore, following sepsis we noticed a significant increase of the activation of caspase-3, the rate-limiting enzyme for apoptosis in the lungs, compared to sham-operated mice. However, the treatment of septic mice with B-1a cells significantly reduced the level of active caspase-3 by mean values of 52%, compared to PBS-treated septic mice (Fig. 4c).

### Treatment with B-1a cells restores IgM levels in lung tissues during sepsis

**Fig. 5:**
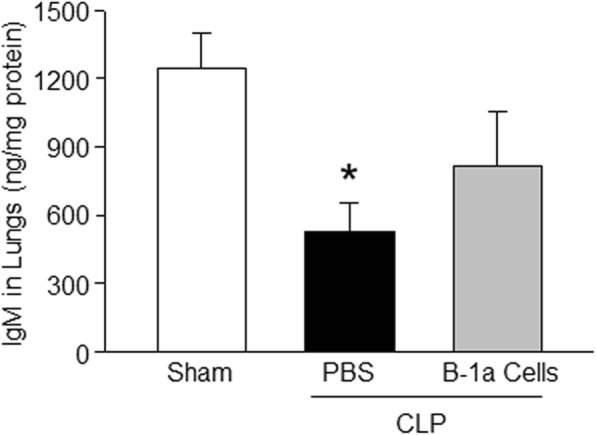
Treatment with B-1a cells increases IgM levels in the lungs following sepsis. A total of 5 × 10^5^ sorted B-1a cells were delivered into the peritoneal cavity of CLP mice. After 20 h, lung tissue was harvested from sham, PBS-, and B-1a cell-treated mice and assessed IgM levels in total extracted proteins by ELISA. Data are expressed as means ± SE (n = 9 mice/group) and compared by one-way ANOVA and SNK method (^*^p < 0.05 vs. sham mice). CLP, cecal ligation and puncture; ELISA, enzyme-linked immunosorbent assay

About 80% of the IgM present in the blood are natural IgM which comes from the B-1a cells and its levels are high at steady-state (Aziz et al. 2015). We previously showed that during sepsis the circulatory (blood) level of IgM were decreased during sepsis, while after adoptive transfer of B-1a cells in the septic mice increased the level of IgM in the blood (Aziz et al. 2017). In addition to this, within the peritoneal cavity (local infectious foci) the IgM levels were also increased following treatment of septic mice with B-1a cells (Aziz et al. 2017). To know whether or not IgM is present in the lungs and their levels are altered during sepsis, we assessed IgM levels in the lung tissues in sham and CLP-operated vehicle- or B-1a cell-treated mice. We found that during sepsis IgM levels in the lungs were significantly decreased as compared to sham mice, while treatment of CLP mice with B-1a cells significantly increased the level of IgM in the lungs (Fig. 5). Therefore, the B-1a cell-mediated protection against sepsis-induced ALI could be mediated through both systemic and local increase of IgM.

### Deficiency of B-1a cells in CD19^−/−^ mice exacerbates lung injury

**Fig. 6:**
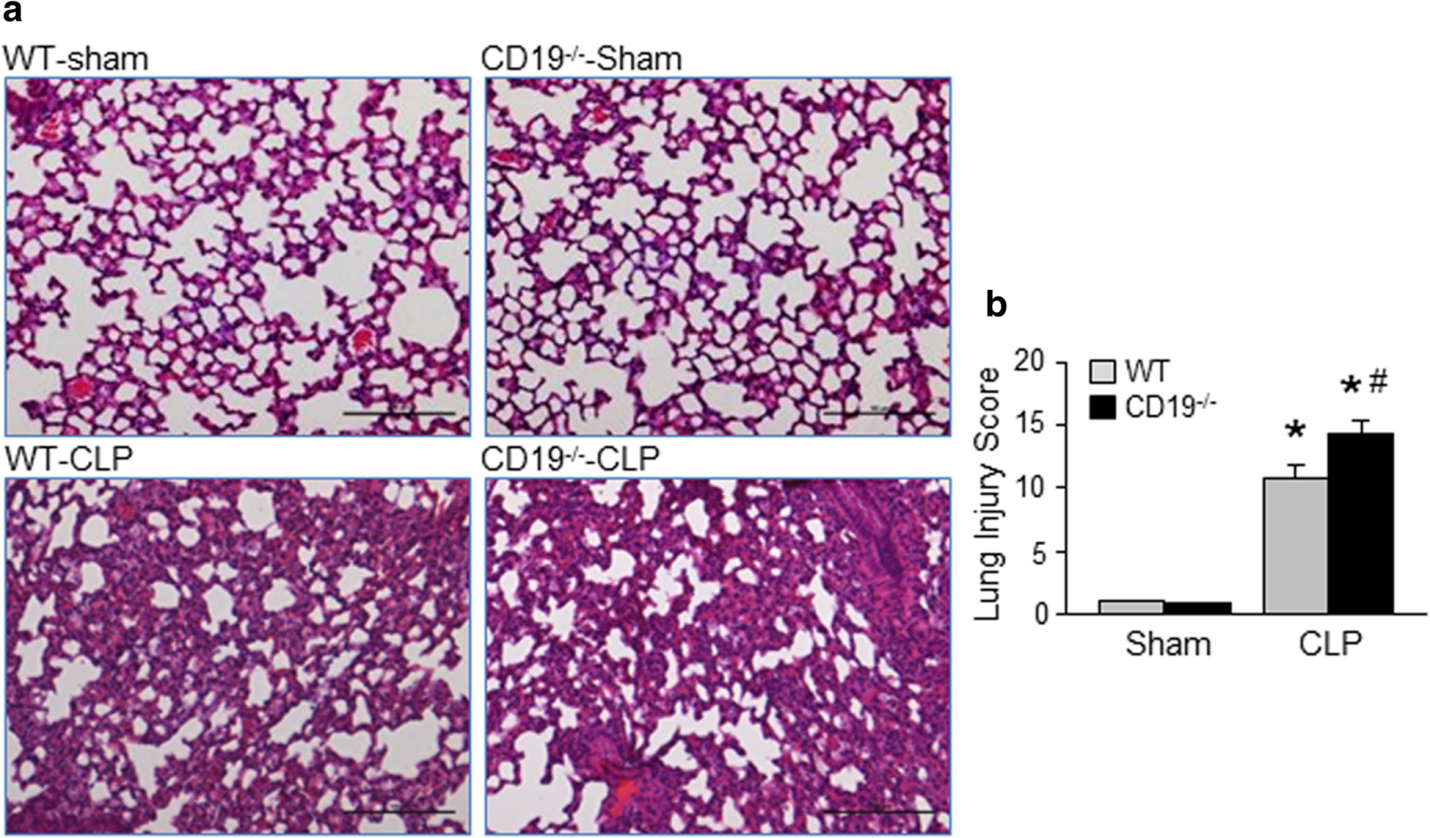
Deficiency of B-1a cells exaggerates lung injury during sepsis. **a** After 20 h of CLP induced in WT and CD19^−/−^ mice, lung tissues were harvested and stained with H&E. The slides were observed under light microscopy at × 100 original magnification in a blinded fashion. Representative images for each group are shown. Scale bar, 100 μm. **b** Histological injury scores of the lungs in WT and CD19^−/−^ mice were quantified as described in Materials and Methods. Data obtained from three independent experiments are expressed as means ± SE (*n* = 6 mice/group) and compared by one-way ANOVA and SNK method (^*^p < 0.05 vs. shams; ^#^*p* < 0.05 vs. WT CLP mice). CLP, cecal ligation and puncture; H&E, hematoxylin and eosin

B cells express the co-receptor CD19, which serves as a positive regulator of B cell receptor (BCR) signaling and is critical for B cell development and activation (Aziz et al. 2017; Aziz et al. 2015; Haas et al. 2005). It has been shown that transgenic mice over expressing CD19 generate excess B-1a cells which provide protection against infection, while CD19-deficient mice lack B-1a cells and are susceptible to infection (Haas et al. 2005). We examined CD19^−/−^ mice to determine whether or not the deficiency of B-1a cells would exacerbate lung injury during sepsis. Following CLP, histological images of the lung tissues showed increased levels of alveolar congestion, exudate, interstitial and alveolar cellular infiltrates, intra-alveolar capillary hemorrhages, and extensive damage of epithelial architecture in CD19^−/−^ mice as compared to WT mice (Fig. 6a). These histological changes were reflected in a significant increase in lung tissue injury score in CD19^−/−^ mice compared to WT mice by a mean value of 54% after CLP (Fig. 6b).

## Discussion

B-1a cells are part of innate immune system and exhibit unique phenotypic, developmental, localizations, signaling and functional characteristics that differ from the conventional B-2 cells (Aziz et al. 2015). B-1a cells are innate-like, while B-2 cells are adaptive-type immune-reactive lymphoid cells. B-1a cells spontaneously secrete germline-like, polyreactive natural antibody (IgM), which acts as a first line of defense by neutralizing a wide range of pathogens (Aziz et al. 2015; Grönwall et al. 2012). B-1a cells are known to produce several immunomodulatory molecules either spontaneously or in the presence of stimulation, which attenuate infectious and inflammatory diseases including influenza, pneumonia, atherosclerosis, inflammatory bowel disease, autoimmunity, obesity and diabetes mellitus [reviewed in (Aziz et al. 2015)]. Recently, the beneficial role of B-1a cells in sepsis has been reported (Aziz et al. 2017; Rauch et al. 2012), and this was shown to be mediated through the control of excessive systemic inflammation and bacterial burdens. Nonetheless, the role of B-1a cells in mitigating inflammation and injuries to the remote organs especially lungs, during sepsis was not known. In the current study, we primarily focused on the role of B-1a cells in attenuating ALI during sepsis.

Using a mouse model of sepsis, we previously showed that the numbers of B-1a cells in peritoneal cavity, spleen and bone-marrow were significantly decreased (Aziz et al. 2017). Adoptive transfer of syngeneic B-1a cells into septic mice significantly attenuated systemic inflammatory and injury parameters as well as bacterial burden in the blood and peritoneal cavity (Aziz et al. 2017). In the current study, we found that the adoptive transfer of murine B-1a cells into septic mice significantly attenuated the expression of pro-inflammatory cytokines IL-6 and IL-1β in the lungs. We also found overall improvement of lung injury scores in B-1a cell-treated mice during sepsis. The attenuation of sepsis-induced lung injury was correlated with reduced levels of chemokine expression, neutrophil infiltration as assessed by MPO, and cellular apoptosis through the down-regulation of caspase-3 activity. We previously demonstrated B-1a cell–deficient CD19^−/−^ mice were more susceptible to infectious inflammation, thereby causing an increased mortality rate in sepsis (Aziz et al. 2017). Here, we also found that the CD19^−/−^ mice showed significantly increased levels of lung injury scores as compared to WT mice after sepsis, thus suggesting the pivotal beneficial role of B-1a cells to protect mice from ALI during sepsis. The improvement of systemic inflammation and lung injury and inflammation after administration of B-1a cells in septic animals can be better reflected in their survival outcomes. In our previous study, we demonstrated significant improvement of the survival outcome in B-1a cell-treated mice over that of PBS-treated mice with sepsis (Aziz et al. 2017). By contrast, the B-1a cell deficient CD19^−/−^ mice had significantly reduced rate of survival as compared to the WT mice during sepsis (Aziz et al. 2017).

The crosstalk effect between B-1a cells and macrophages has been demonstrated in previous reports (Thies et al. 2013; Barbeiro et al. 2011). B-1a cells produce IL-10 in response to LPS stimulation (Aziz et al. 2017; Barbeiro et al. 2011). In B-1a cells and macrophages co-cultures, production of pro-inflammatory cytokines was lower and the production of anti-inflammatory cytokine IL-10 was higher than in macrophage monocultures (Barbeiro et al. 2011). Interestingly, co-culture of IL-10^−/−^ B-1a cells and WT macrophages did not reduce the levels of the pro-inflammatory cytokines (Aziz et al. 2017), indicating the pivotal regulatory role of B-1a cells in controlling inflammation. Beside these in vitro findings, we demonstrated the beneficial role of B-1a cells during sepsis through the production of anti-inflammatory cytokine IL-10 (Aziz et al. 2017). Lungs contain resident alveolar macrophages which during sepsis become activated to produce excessive amounts of pro-inflammatory cytokines and chemokines (Aziz et al. 2012; Moldoveanu et al. 2009). However, we noticed significant decreases in the expression of pro-inflammatory cytokines IL-6 and IL-1β and chemokine MIP-2 in the lungs of B-1a cell-treated mice during sepsis. Since B-1a cells are known to produce excessive amounts of anti-inflammatory cytokine IL-10, it is therefore understandable that the B-1a cells could temper the pro-inflammatory responses of alveolar macrophages and thus protect mice from ALI during sepsis. B-1a cells can serve as antigen presenting cells, providing effective signaling to T-cells via CD80 and CD86 molecules, which are expressed on B-1a cells (Aziz et al. 2015). Therefore, in parallel to study the crosstalk effect between B-1a cells and macrophages, it would be of interest for future studies to elucidate the novel role of B-1a cells on T cells in the lungs during sepsis.

GM-CSF is mainly produced by the innate response activator (IRA) B cells (Rauch et al. 2012). Our current study focused on the effect of IL-10- and IgM-producing B-1a cells in sepsis-induced ALI. In our previous study, we demonstrated that the septic mice treated with IL-10^−/−^ B-1a cells did not show protection against sepsis (Aziz et al. 2017), thus pointing to the role of B-1a cell-secreted IL-10 to exert beneficial role in sepsis. We also demonstrated that the levels of GM-CSF in B-1a cells between WT and IL-10^−/−^ mice strains following sepsis were remained same (Aziz et al. 2017), indicating that the lack of IL-10 in B-1a cells could be detrimental in sepsis without affecting the levels of GM-CSF. Future studies focusing on the role of GM-CSF producing IRA B cells will help reveal the importance of IRA B cells in sepsis-induced ALI.

In sepsis, irresistible migration of neutrophils into the lungs leads to endothelial cell injury and sustained inflammation (Aziz et al. 2013; Aziz et al. 2012; Hirano et al. 2015; Hirano et al. 2016). The patients with ARDS represent huge infiltration of neutrophils in the lung tissues which correlates with the severity of lung injury as a result of releasing ample amounts of proteolytic enzymes and pro-inflammatory mediators from the infiltrated neutrophils into the lung tissue beds (Abraham 2003; Williams and Chambers 2014). Thus, it is suggested that the regulation of neutrophil infiltration into the lungs could be an effective therapeutic approach in septic-induced ALI. Here, in the current study, we noticed dramatic reduction of neutrophil infiltration in the lungs as measured by MPO and chemokine MIP-2 levels which ultimately led to diminished lung tissue injury in the B-1a cell-treated mice. Although the direct roles of B-1a cells on macrophages and T cells had been delineated previously, the effect of B-1a cells on neutrophils is largely unknown. Elucidation of the direct role of B-1a cells on neutrophils will provide additional insights into the pathophysiology of ALI in sepsis.

In the context of lung injury and inflammation caused by viral and bacterial infections, several reports have already demonstrated the beneficial role of B-1a cells in protecting mice from lung injury, mainly mediated through the release of natural IgM (Baumgarth et al. 1999; Baumgarth et al. 2000; Weber et al. 2014). Natural IgM secreted from B-1a cells eliminates invading pathogens and also scavenges dying cells, which in turn can attenuate inflammation and tissue injury (Grönwall et al. 2012; Vas et al. 2012). On the other hand, mice lacking natural IgM are prone to develop autoimmune diseases because of the failure to neutralize/remove antigens and apoptotic cells to maintain homeostasis (Aziz et al. 2015; Boes et al. 2000). In the current study, we noticed significant reduction in the number of apoptotic cells in the lungs following B-1a cell treatment in septic mice. Although here we did not assess the phagocytic clearance of apoptotic cells by professional phagocytes, we found that the septic mice treated with B-1a cells showed reduced levels of caspase-3 activity, indicating inhibition of cellular apoptosis by B-1a cell treatment. It has been demonstrated that endothelial cell pyroptosis, a form of cell death, may result in sepsis-induced ALI through the activation of caspases (Cheng et al. 2017; Aziz et al. 2014). Since the pyroptotic cells also undergo DNA fragmentation and, like apoptotic cells show positive TUNEL staining (Mariathasan et al. 2005), our TUNEL assay data in lung tissues pointed to the possibility of decreased pyroptosis of lung cells following treatment of septic mice with B-1a cells. Further studies by staining the lung tissue sections with endothelial cell marker CD31 Ab, TUNEL and caspase-1 Ab will help confirm the status of endothelial cell pyroptosis in lungs during sepsis, and also demonstrate the inhibitory effect of B-1a cells for endothelial cell pyroptosis during sepsis.

During influenza virus infection, the therapeutic potential of murine B-1a cells was mainly generated by their enrichment at the lungs as a result of their translocation from serosal cavities where they are generally localized at the steady-state condition (30). Following their translocation into lungs, B-1a cells autonomously secrete natural Abs and other immunomodulatory molecules to protect hosts against influenza virus infection (Aziz et al. 2015; Baumgarth et al. 1999; Baumgarth et al. 2000). In line with this fact, Weber, et al*.* showed B-1a cells migrate from the pleural cavity to the interstitial lung tissues, where they produce ample amount of GM-CSF and natural Abs to protect the host from endotoxin or *S. pneumoniae*-induced ALI in mice (Weber et al. 2014). In the current study utilizing murine model of sepsis, B-1a cells could be enriched into the lungs as a result of their translocation from the site of origin to protect mice against lung inflammation.

In the current study, we injected septic mice with B-1a cells at the time of CLP operation, the post-treatment of septic mice with B-1a cells would help advance our current therapeutic strategy towards more clinically relevant circumstances. We basically chose to treat mice with B-1a cells immediately after CLP rather than post-surgery because most of the pro-inflammatory cytokines and chemokines are expressed early/hyperdynamic phase in sepsis, reaching maximum levels around 10–12 h after CLP and then returns to normal levels (Aziz et al. 2013; Bosmann and Ward 2013; Rittirsch et al. 2008). Therefore, in order to obtain optimal inhibition of pro-inflammatory cytokines and chemokines by the treatment of B-1a cells, we chose time of treatment at CLP induction instead of a later time point. We delivered the B-1a cells into the septic mice through the intraperitoneal route; however, administration of B-1a cells intravenously would help shift this laboratory strategy to bedside approaches.

In the present study, we used C57BL/6 WT mice, also known as B6 mice obtained from the Taconic to compare the outcomes of sepsis-induced ALI with B6 background B-1a cell deficient CD19^−/−^ mice obtained from the Jackson lab. Our previous studies on B6 background of mice of Taconic and Jackson lab showed similar outcomes in their survival in CLP-induced sepsis (Giangola et al. 2013; Qiang et al. 2013). However, since the immune responses of mice may vary among various strains and vendors (Otto et al. 2016), we consider this as one of our limitations in experimental designing. Further studies using control WT mice and CD19^−/−^ mice from the same vendor will strengthen our present finding of the beneficial effect of B-1a cells on ALI during sepsis.

## Conclusions

We identified the beneficial role of murine B-1a cells in sepsis-induced ALI through the mitigation of inflammation and injury to the lungs. Recently, a B cell population in human has been identified which represents functional characteristics that match with murine B-1a cells, including autonomous production of natural IgM, constitutive basal expression of intracellular signal transduction molecules, and effective stimulation of T lymphocytes (Aziz et al. 2015; Griffin et al. 2011; Rothstein et al. 2013). Our current study demonstrating the role of mouse B-1a cells in sepsis-induced ALI further focuses on identifying valuable lessons that may be applicable to human B-1a cells.

## Abbreviations

ALI: Acute lung injury
ARDS: Acute respiratory distress syndrome
BCR: B-cell receptor
CLP: Cecal ligation and puncture
ELISA: Enzyme-linked immunosorbent assay
FO: Follicular
GM-CSF: Granulocyte-macrophage colony-stimulating factor
LPS: Lipopolysaccharides
MIP-2: Macrophage-inflammatory protein-2
MPO: Myeloperoxidase
MZ: Marginal zone
NETs: Neutrophil extracellular traps
NO: Nitric oxide
PAMP: Pathogen-associated molecular pattern
PBS: Phosphate-buffered saline
ROS: Reactive oxygen species
TLR: Toll-like receptor
TUNEL: Terminal deoxynucleotide transferase dUTP nick end labeling
